# Negotiating household heat: thermal labor, energy justice, and women’s health in Nepal’s Madhesh Province

**DOI:** 10.3389/fpubh.2025.1657267

**Published:** 2025-07-24

**Authors:** Animesh Ghimire, Mohan Das Manandhar, Sarita Karki, Karuna Bajracharya

**Affiliations:** 1 Sustainable Prosperity Initiative Nepal, Kathmandu, Nepal; 2 Clean Cooking Alliance, United Nations Foundation, Washington, DC, United States

**Keywords:** climate change, heat stress, cooking, biomass, adverse effects, women’s health, health equity, policy

## Abstract

**Introduction:**

Household cooking with solid fuels exposes women to prolonged indoor heat levels that routinely exceed internationally accepted occupational safety thresholds; yet, this exposure remains largely absent from climate-health analyses. This perspective article introduces the concept of thermal labor—the physiological strain, time cost, and health risks associated with performing domestic work under chronically elevated kitchen temperatures—and argues that such exposure constitutes an overlooked driver of gendered health inequities in Nepal’s Madhesh Province.

**Methods:**

Evidence was synthesized from national temperature records, caste-disaggregated census data, spot measurements conducted by the Nepal Health Research Council, and illustrative intervention studies from South Asia and Africa. The policy context was examined through Nepal’s Nationally Determined Contribution, the Clean Cooking Alliance Nepal Country Action Plan, and the National Disaster Risk Legislation.

**Results:**

The synthesis suggests that accelerated warming in Nepal’s lowlands and caste-linked reliance on biomass fuels result in daily indoor heat exposures. Prior studies associate such exposures with appetite suppression, reduced dietary diversity, and increased time burdens for women who manage household cooking. These established pathways, when considered alongside the socioeconomic profile of Dalit households in Madhesh, indicate a heightened but under-documented risk for this group. Nepal’s existing target of achieving electric cooking adoption in 31.5 percent of households by 2035 offers a practical policy lever for reducing thermal exposure and its associated health and equity impacts.

**Discussion:**

Positioning thermal labor as a measurable health determinant broadens the clean-cooking agenda beyond smoke reduction to encompass heat mitigation, nutrition, and gender equity. A balanced approach is proposed: sentinel kitchen-heat surveillance within existing household surveys would establish exposure baselines; thermal-performance criteria in stove-procurement standards could translate policy commitments into verifiable outcomes; and integrating heat indicators into clean-cooking and disaster-risk frameworks would facilitate coordinated action. These steps would convert domestic heat from an invisible stressor into a tractable public health target, illustrating how a single intervention pathway can advance climate, energy, and equity goals.

## 1 Introduction

Exposure to extreme heat is increasingly recognized as a major climate-related threat to population health. Nonetheless, empirical research and policy guidance continue to focus primarily on episodic heat-wave mortality in urban centers and on productivity losses among outdoor laborers in construction and agriculture (1, 2). A parallel, chronic exposure—high temperatures generated within homes where an estimated 2.4 billion individuals cook with solid fuels—receives comparatively limited attention (3). Combustion in open fires and rudimentary stoves emits not only particulate pollution but also sustained radiant and convective heat that elevates kitchen temperatures far above safe physiological limits (4). Because this exposure occurs in the domestic sphere and is borne predominantly by women, it is seldom quantified, rarely regulated, and almost never classified as an occupational hazard.

Thermal labor, as used in this article, refers to the combined physiological strain and time demand incurred when domestic work is performed in kitchens whose operative temperatures consistently exceed internationally recommended heat-stress limits. Although the term could theoretically apply to any household task conducted in unsafe thermal conditions, this study concentrates on cooking because (i) it generates the most intense and sustained indoor heat and (ii) it occupies the largest share of rural Nepali women’s domestic time budget. Time-use studies conducted in rural Nepal show that women spend about four hours per day cooking on traditional biomass stoves and an additional 374 h per year collecting firewood (5, 6). Firewood gathering, though performed outdoors, imposes a separate time burden that indirectly perpetuates kitchen heat by sustaining reliance on biomass fuels.

This dual burden disproportionately impacts Dalit women, considered untouchables based on caste, who often live with their families in single-room dwellings and face deep-rooted barriers to modern energy, credit, and local governance (7, 8). By situating thermal labor at the intersection of caste, gender, and climate vulnerability, the present analysis shows how rapid warming, structural discrimination, and energy poverty converge in Madhesh Province to intensify household heat exposure. The article concludes with research and policy proposals aimed at converting thermal labor from an invisible burden into a measurable public-health indicator and tractable policy target.

## 2 Nepal’s evolving thermal landscape

Nepal, with 29.6 million inhabitants, extends approximately 193 km from the subtropical lowlands (elevations <100 m) to the crest of the Himalaya, where Mount Everest rises to 8,849 m (9, 10). This marked altitudinal range creates strong climate gradients, yet instrumental records demonstrate warming across all zones. Mean annual temperature has increased by 0.06°C per year since the late 1970s; in the high mountains, the rate already exceeds the global average (11).

**Figure 1:**
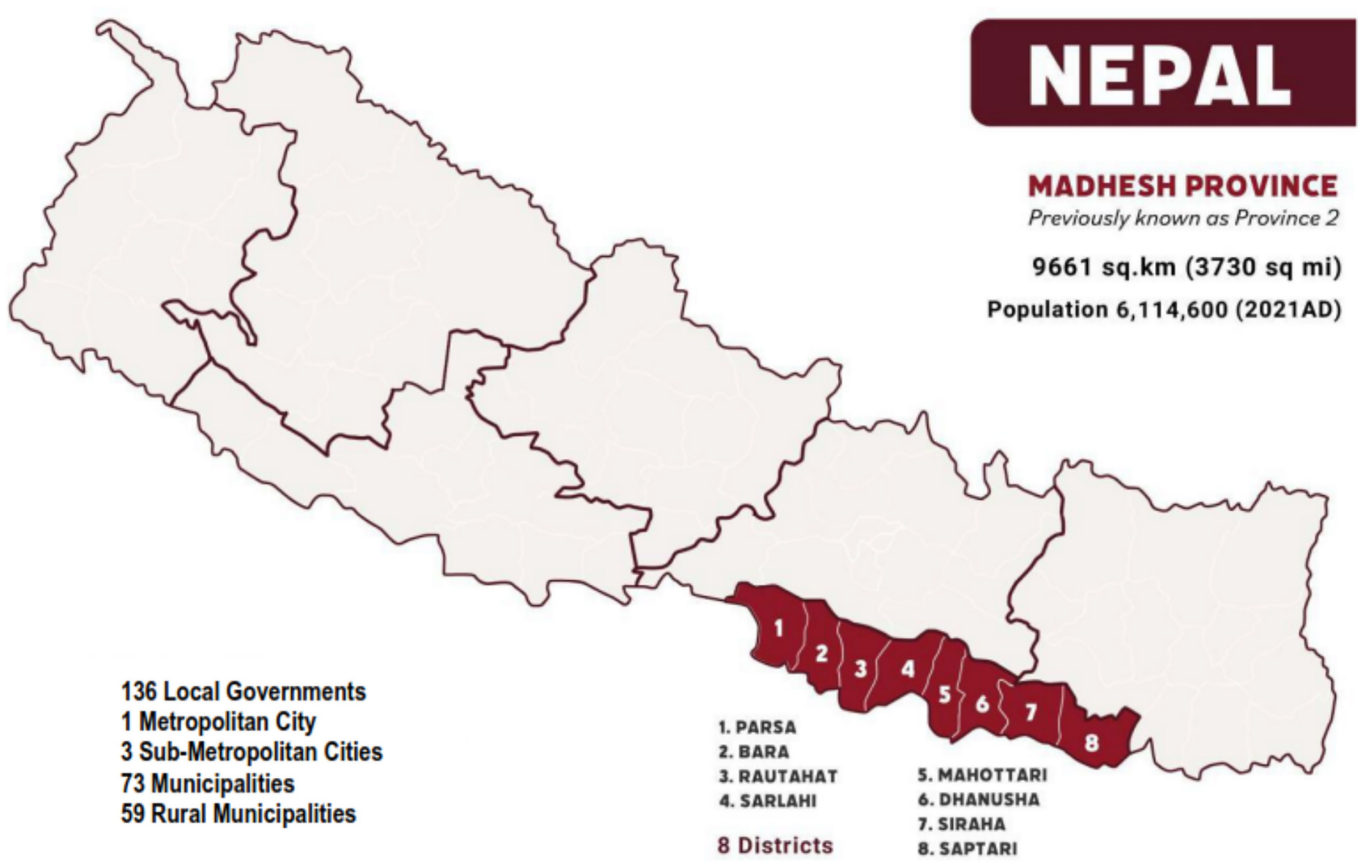
Madhesh Province Map and Data (14).

The southern Terai lowlands occupy about 23% of Nepal’s land area but support 54% of its population (12). Within the eastern Terai lies Madhesh Province (Figure 1), a 400-km corridor along the Indian border that functions as the country’s principal grain basket. Although it constitutes only 6.5% of the national territory, the 2021 census recorded 6.1 million residents, making Madhesh the most densely populated of Nepal’s seven provinces (13, 14). Summer daytime temperatures commonly exceed 40°C, and analyses by the Nepal Health Research Council (NHRC) identify Janakpur, the provincial capital, as the fastest-warming meteorological station in the country (15).

Modeling studies highlight significant public-health implications. Under a high-emissions scenario and in the absence of targeted adaptation, heat-related mortality in Nepal is projected to rise from an estimated 4 to more than 50 deaths per 100,000 population per year by 2080 (15). Because national surveillance systems primarily monitor outdoor workers (16), these projections understate risks for women who spend prolonged periods near household stoves.

Socioeconomic conditions further increase vulnerability. Madhesh exhibits both the highest poverty incidence and the most pronounced caste stratification among the provinces (12). Dalits, historically marginalized within the Hindu caste hierarchy, constitute approximately 19% of the provincial population (17). Caste-disaggregated census microdata show that only 4% of Dalit households in the Terai use clean cooking fuels, compared with 31% of non-Dalit households (4). Firewood, dung, and crop residues therefore dominate Dalit energy portfolios, and cooking is typically performed in single-room, clay-walled dwellings with limited ventilation (18).

The NHRC heat wave report does not report indoor/kitchen temperature monitoring; rather, it characterizes heat severity using heat index categories (apparent temperature), classifying 41–54°C as “Danger” and >54°C as “Extreme danger” (15). For comparison, the International Labour Organization recommends maintaining a wet-bulb globe temperature below 28°C for continuous heavy work; values above 30°C require work–rest cycles to prevent heat strain (19). These regional heat index levels, therefore, exceed established occupational-safety thresholds by a substantial margin, rendering dehydration, renal stress, high heart rate, and blood pressure plausible daily outcomes rather than isolated events (20–22).

## 3 From indoor air pollution to thermal labor

Scholarship on household energy has traditionally focused on particulate matter and carbon monoxide—a concentration warranted by an estimated 3.2 million premature deaths each year attributable to household air pollution (23). The same combustion sources, however, generate a second and largely independent exposure pathway: sustained heat. Recognizing this thermal load reframes cooking as labor performed in a micro-environment that, in occupational health terms, approximates an unregulated high-temperature workplace.

We employ the term *thermal labor* to denote the physiological strain, time cost, and health risk incurred when domestic tasks are undertaken under elevated thermal conditions. This concept extends Batliwala’s seminal characterization of women’s “rigorous imprisonment at the hearth” (24) by incorporating contemporary evidence from heat-health science.

The medical literature links chronic heat exposure to dehydration, acute kidney injury, impaired cognitive performance, and adverse pregnancy outcomes (25). While such associations are well documented for agricultural and construction workers, analogous risks within the household remain understudied. In the Nepal Health Research Council’s 2018 heat-risk survey of outdoor laborers in the Terai, 58 percent reported at least one heat-related illness episode in the preceding 12 months; the most common symptoms were dizziness, decreased appetite, and fatigue (15). These manifestations are equally plausible among women who spend several hours each day beside biomass stoves, yet they are rarely captured in routine clinical data or national surveillance systems.

By foregrounding thermal labor, this article seeks to broaden the health and equity discourse around household energy. Doing so not only complements existing efforts to reduce smoke-related morbidity but also highlights a modifiable exposure with direct implications for women’s productivity, nutritional practices, and long-term well-being.

## 4 Intersectionality: gender, caste, and poverty

Applying an intersectional framework illuminates why Dalit women in Madhesh bear the heaviest cumulative burden of household heat. Gender norms designate women as primary food preparers; household poverty constrains the adoption of liquefied-petroleum gas (LPG) or electric stoves; and caste-based exclusion limits access to credit, land, and representation in local governance (26). Ethnographic studies consistently locate Dalit women at the convergence of the longest cooking hours, the most distant fuel-collection routes, and the lowest levels of intra-household bargaining power (18).

A 2025 Mercy Corps assessment of 347 public schools in Madhesh Province found that 91 percent of schools recorded at least one heat-related illness episode during the previous academic year 2023/24 (27). Heat also disrupted learning through both closures and absenteeism. Schools shut for an average of 10.9 days per year following Department of Hydrology and Meteorology heat-wave alerts, with some remaining closed for as long as 41 days. Among those that stayed open, one-third shifted to morning classes as an adaptive measure.

Crucially, the survey documented marked gender differences in attendance: girls missed 29 days and boys 25.0 days, on average, owing to extreme heat and heat-related illness. Focus-group discussions confirmed that parents keep daughters home more frequently, citing both health concerns and the need for additional household labor. These data strengthen the argument that household-level thermal burdens spill over into the education sector, disproportionately curtailing girls’ learning opportunities and, by extension, their long-term earning potential. Interventions that cool domestic environments—alongside school-based heat-adaptation measures—are therefore pivotal for safeguarding both immediate health and gender-equitable human-capital development in marginalized communities.

## 5 Thermal labor, nutrition, and time poverty

**Figure 2:**
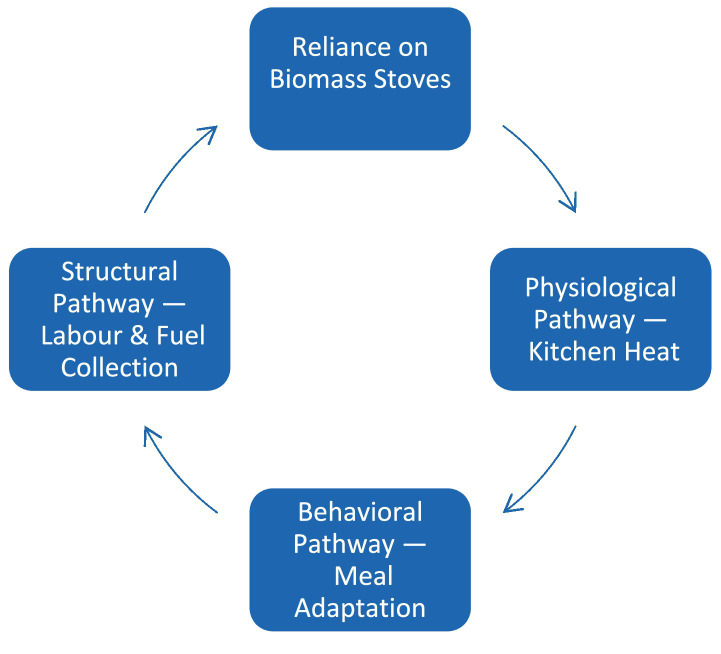
Reinforcing feedback loop linking biomass reliance to physiological, behavioral, and structural heat-stress pathways. Starting at the top, continued reliance on biomass stoves (Node 1) elevates indoor temperatures, generating the physiological pathway—kitchen heat (Node 2). Direct thermal strain can suppress appetite and fluid intake. Elevated heat concurrently triggers the behavioral pathway—meal adaptation (Node 3), in which households alter their dietary preferences, tending toward staples of lower nutrient density. Prolonged cooking and year-round fuel gathering constitute the structural pathway—labor and fuel collection (Node 4); this workload sustains dependence on biomass fuels and closes the loop back to Node 1. Each arrow denotes a positive (reinforcing) influence: increases in any node intensify the next, collectively maintaining high kitchen heat and associated nutrition risks.

The 2022 Nepal Demographic and Health Survey documents that 24.8 percent of Nepali children under five are stunted, with the prevalence rising to 29 percent in Madhesh Province (28). Conventional explanations emphasize inadequate diet, infectious disease, summer heatwaves, and household poverty among others (29, 30). These stressors are acute in Madhesh, which records the country’s lowest Human Development Index out of the seven provinces, with a score of 0.519 (31). Building on this context, our analysis identifies an additional household-level contributor: the intense heat generated by biomass cooking We posit that this thermal burden undermines nutrition through three mutually reinforcing pathways (Figure 2):

1. Physiological — cooking zone heat exposure suppresses appetite and can reduce fluid intake;
2. Behavioral — to limit exposure, households shift toward less-diverse dietary preferences; and
3. Structural — the labor required for cooking and year-round fuel collection entrenches reliance on biomass stoves, perpetuating indoor heat.

### 5.1 Physiological pathway

Field reports by the Nepal Health Research Council indicate that regional midsummer heat indices reach “Danger” (41–54°C) and “Extreme danger” (>54°C) levels (15). At such extremes, even modest additional heat loads become physiologically consequential: controlled laboratory studies demonstrate that a 1°C rise in core body temperature can reduce voluntary food intake by 10–15 percent (32). The compounding thermal burden of cooking in these extreme ambient conditions, therefore, constitutes a clinically meaningful threat to appetite, hydration, and overall metabolic balance. Conceptual work on thermal ecology corroborates this mechanism; Youngentob et al. (33) reports that elevated ambient heat consistently suppresses voluntary feeding, reinforcing appetite suppression as a credible link between household heat and undernutrition. As Youngentob’s synthesis is mainly derived from controlled ecological settings rather than an indoor kitchen environment, it should be viewed as illustrating a general biological principle; empirical studies that couple continuous kitchen-temperature monitoring with dietary-intake surveys in Madhesh are still required to quantify the magnitude of this effect in real-world conditions.

On the other hand, thermal vulnerability varies across populations. Children and older adults have weaker thermoregulatory systems, and preexisting health conditions increase their risk of heat-related loss of appetite and dehydration (34). Gender further modulates risk: women, on average, exhibit lower heat-tolerance thresholds than men (35). Social norms then compound this physiological disparity— a global analysis indicates that women prepare 4.3 more meals per week than men (36). These findings suggest that sustained kitchen temperatures may directly reduce dietary intake, with disproportionate impacts on those already carrying the heaviest domestic workloads.

### 5.2 Behavioral pathway

Studies from low-income settings demonstrate that high kitchen temperatures, coupled with limited fuel budgets, shape day-to-day dietary decisions. In Nairobi’s informal settlements, women report avoiding dishes that require prolonged simmering or large quantities of fuel because poorly ventilated kitchens become intolerably hot during cooking (37). Households therefore substitute refined cereals or purchase ready-to-eat street foods, sacrificing nutrient density for thermal comfort and fuel savings. Similar patterns are observed in Kampala, Uganda, where cash-constrained families deliberately eschew energy-intensive recipes in favor of staples that minimize stove use and heat build-up (38). Region-wide evidence reinforces this response: an Asian survey found that 89 percent of respondents were dissatisfied with their kitchen thermal environment and that discomfort discouraged dishes needing extended preparation (39).

Although these studies were conducted outside Nepal, they converge on a consistent behavioral trajectory: when cooking raises indoor temperatures to uncomfortable levels, households simplify food choices, thereby narrowing dietary diversity. Given the severe compounding thermal burden of cooking during midsummer heatwaves, a comparable risk of heat-driven dietary simplification likely exists in Madhesh. The phenomenon is therefore treated here as a plausible—but not yet empirically verified—mechanism linking kitchen heat to nutrient shortfalls.

### 5.3 Structural pathway

Socio-demographic profiles help identify groups most exposed to heat stress. Low-income rural households have limited access to clean fuels, mechanical ventilation, and adaptive health services; consequently, they rely on traditional biomass stoves that intensify kitchen temperatures. Within these households, Nepali women shoulder the bulk of cooking and fuel procurement, spending about 4 h each day preparing food and approximately 374 h gathering firewood annually (5, 6). Although fuel collection occurs outdoors, the associated time cost entrenches biomass reliance and, by extension, sustained indoor heat exposure.

Introducing higher-efficiency stoves can disrupt this cycle. In rural Kenya, adoption of the Kuniokoa stove reduced women’s active cooking time by 70 min per day and fuel-collection time by 5 h per week (40). Although the trial recorded no temperature data, post-intervention interviews noted a perceptible drop in radiant heat—a feature also highlighted in user-centered design studies from Indonesia (41). These qualitative data support Diagnestya’s (41) broader argument that open fire cooking imposes a substantial thermal burden that limits both productive labor and dietary variety. Conversely, studies show that improved cookstoves shorten active cooking tasks, lower indoor temperatures, and create conditions more conducive to income-generating activities (22, 42, 43). It remains uncertain, however, how often reclaimed time translates into measurable nutritional or economic gains, given evidence that newly available hours may be absorbed by performing additional household duties or other unpaid tasks (44).

In sum, the structural pathway operates through two inter-locking mechanisms: (i) the labor time that anchors households to biomass fuels and (ii) the elevated indoor thermal environment that amplifies physiological and behavioral risks. Recognizing—and measuring—both components is essential for designing interventions that deliver simultaneous gains in health, nutrition, and women’s economic agency.

## 6 Discussion

### 6.1 Policy implications: measuring and mitigating thermal risk

Most low-and middle-income countries track household air-pollution indicators, but seldom measure household heat. Incorporating a concise thermal-assessment module into Nepal’s forthcoming Demographic and Health Survey would yield a nationally representative baseline at modest additional cost. Enumerators could record two spot kitchen-temperature readings—one during the principal meal and one during a cooler reference period—while documenting stove type and ventilation. Where finer resolution is required, low-cost passive peak-temperature indicators (wax-encapsulated dyes that change color irreversibly above a specified threshold) can be distributed alongside cookstove programs; these single-use devices are battery-free and compatible with local technical capacity (45). Although less detailed than electronic loggers, they can identify households at greatest thermal risk.

Nepal’s Nationally Determined Contribution (NDC) commits to electric cooking in 31.5 percent of households by 2035 (46). To translate this target into measurable heat relief, future stove procurement tenders should include explicit performance criteria, such as maximum external surface temperature, reduction in kitchen air temperature, and independently verified time savings. Achieving these technical standards, however, depends on the resilience of the electricity supply that will power the new appliances. Approximately 90 percent of grid electricity is sourced from hydropower, which is susceptible to glacier-lake outburst floods and monsoon variability (47). Consequently, a two-track strategy is advisable. High-efficiency induction or electric stoves can be prioritized in peri-urban areas where grid stability permits, accompanied by tariff incentives that favor off-peak cooking. In rural Madhesh, where electrification remains uneven, well-insulated improved biomass stoves (48) or solar–electric hybrid models (49) offer improved end-use efficiency, thereby mitigating the overall thermal burden of cooking while accommodating local food practices (50).

Procedural justice is vital for sustained adoption. Programs designed without meaningful participation from women—particularly those from marginalized castes—often experience low uptake. Municipal energy committees in Madhesh should therefore designate voting seats and budget-approval authority for women from these groups, operationalizing Batliwala’s assertion that technologies succeed only when shaped by the individuals they affect (24).

Thermal safety should also be integrated into Nepal’s Disaster Risk Reduction and Management Act. Existing heat-action plans primarily address outdoor laborers, such as street vendors and construction workers (51). Extending midday rest recommendations, hydration campaigns, and SMS-based heat alerts to individuals responsible for domestic cooking would close a critical policy gap. Finally, linking clean-cooking subsidies to child-nutrition initiatives can help ensure that the time saved through cooler kitchens is devoted to preparing more diverse and nutritious meals rather than being absorbed by additional unpaid work.

### 6.2 Practical research priorities

#### 6.2.1 Sentinel exposure surveillance

The forthcoming Demographic and Health Survey could incorporate a sentinel kitchen-heat module targeted at a purposive sample of Madhesh households that continue to rely on solid-fuel stoves. Enumerators would document: (i) kitchen configuration, including degree of enclosure and ventilation dimensions; (ii) peak meal-time air temperature measured once with a handheld infrared thermometer; and (iii) stove-embedded usage analytics generated by the smart induction units currently being deployed in the province, which record burner-on duration and electricity consumption (52). Linking a single temperature reading with 30 days of anonymized stove-use telemetry would provide the first empirically grounded map of household heat exposure and associated time burdens, thereby informing the geographic targeting of clean-cooking interventions and refining exposure–response models.

#### 6.2.2 Behavioral field trials

Field experiments should evaluate financing and design strategies that accelerate household transitions to heat-reducing stoves while tracking shifts in intra-family cooking roles. One option, recently piloted by the Clean Cooking Alliance in Nepal, is a small direct cash-transfer tied to documented use of an LPG or induction stove; the study demonstrated that modest payments can increase clean-fuel utilization and decrease reliance on traditional biomass technologies without provoking household conflict (53). Parallel qualitative work from Kenya shows that user-centric stove design can, on its own, prompt greater male engagement: more than one-third of women adopting the modern Kuniokoa stove reported that husbands or teenage sons voluntarily took over cooking because the appliance was smoke-free, did not require floor-level fire tending, and conferred a sense of modernity (40).

Building on these insights, we propose a ward-randomized trial with three arms: (i) an energy-efficient induction stove plus a time-limited cash rebate conditional on verified use; (ii) the stove alone (no cash incentive); and (iii) a ‘business-as-usual’ group that receives no intervention and continues its current cooking practices (control). Objective endpoints will include percentage displacement of biomass use (smart-meter data) and changes in male stove-use minutes. Weekly brief surveys will record perceived fairness and any domestic tension, ensuring that role shifts occur without adverse social consequences. By triangulating financial and design levers, the trial will identify the least-cost, culturally acceptable pathway to reduce women’s cumulative heat exposure.

#### 6.2.3 Nutrition–energy linkage pilots

Six-month community pilots, implemented in collaboration with existing maternal and child nutrition programs, should pair improved biomass or electric stoves with targeted nutrition counseling. End points would include household Dietary Diversity Scores and women’s Minimum Dietary Diversity at baseline and follow-up, thereby testing whether cooler kitchens translate into measurable dietary gains and addressing appetite-suppression mechanisms identified by Mirzabaev et al. (54).

#### 6.2.4 Resilient electrification modeling

Using load-flow data from the Nepal Electricity Authority, researchers should model the least-cost combination of grid extension, solar mini-grids, and advanced biomass or induction stoves that simultaneously minimizes greenhouse-gas emissions and household heat exposure (55). Scenario parameters should incorporate the hydropower climate-risk factors described by Gunatilake et al. (47), including glacier-lake outburst probability and monsoon variability, to ensure that recommendations remain robust under plausible future hydrological conditions.

Collectively, these research activities leverage existing survey infrastructure, short-cycle pilot designs, and readily available administrative data, providing decision-makers with actionable evidence at a cost considerably lower than that of large, standalone studies.

## 7 Limitations

This article synthesizes data from national surveys, small-scale field investigations, and gray literature to argue that domestic thermal exposure is a critical yet underrecognized public health concern. Several factors limit the robustness and generalizability of the analysis. First, peak kitchen-temperature estimates for Madhesh Province are based on single-point measurements; continuous, population-representative monitoring is unavailable, creating uncertainty regarding seasonal and diurnal exposure patterns. Second, causal linkages between thermal exposure, nutritional status, and time poverty are inferred from cross-sectional associations and intervention studies; experimental evidence specific to Nepal is lacking. Third, projected heat-related mortality relies on scenario models that are sensitive to assumptions about future emissions trajectories and adaptive capacity, both of which may evolve with new policies or technologies. Fourth, although gender and caste are central to the discussion, other intersecting dimensions of vulnerability—such as age, disability, and migration status—are not systematically analyzed, which may underestimate the scope of at-risk populations. Finally, the operational feasibility of the proposed surveillance tools and stove procurement criteria has not yet been demonstrated in Nepal, highlighting the need for pilot testing before programmatic scale-up. Acknowledging these limitations highlights the importance of conducting targeted primary research and engaging in iterative policy experimentation to refine, validate, and implement the strategies outlined in this article.

## 8 Conclusion

Domestic thermal exposure lies at the nexus of climate adaptation, gender equity, and energy security. Conceptualizing the heat produced by biomass cooking as thermal labor—a quantifiable health and economic burden—reveals a policy lever capable of reducing greenhouse-gas emissions, alleviating women’s time poverty, improving household nutrition, and enhancing the bargaining power of Dalit women. Integrating straightforward heat indicators into Nepal’s clean-cooking roadmap, Nationally Determined Contribution, and Disaster Risk Reduction Act would transform an invisible stressor into a monitored and budgeted public health target.

The evidence reviewed in this article indicates that postponing action perpetuates preventable morbidity, educational deficits, and intergenerational poverty. The policy question is therefore not whether domestic kitchens should be cooled, but how expeditiously a suite of electric, solar-assisted, and advanced biomass technologies can be deployed under governance structures that prioritize women’s decision-making. Timely implementation would position Nepal as a model for integrated climate-health policy, whereas continued delay would extend today’s thermal burden to future generations.

## Data Availability

The original contributions presented in the study are included in the article/Supplementary material, further inquiries can be directed to the corresponding author.
