# Correction: Negotiating household heat: thermal labor, energy justice, and women's health in Nepal's Madhesh Province

**DOI:** 10.3389/fpubh.2026.1833094

**Published:** 2026-05-08

**Authors:** Animesh Ghimire, Mohan Das Manandhar, Sarita Karki, Karuna Bajracharya

**Affiliations:** 1 Sustainable Prosperity Initiative Nepal, Kathmandu, Nepal; 2 Clean Cooking Alliance, United Nations Foundation, Washington, DC, United States

**Keywords:** climate change, heat stress, cooking, biomass, adverse effects, women's health, health equity, policy

Text originally stated: “NHRC field measurements have documented kitchen air temperatures of 50–54 °C (122–129 °F) during midsummer meal preparation in such dwellings [15]”.

A correction has been made to the section **2 Nepal's evolving thermal landscape**, Paragraph 5:

“The NHRC heat wave report does not report indoor/kitchen temperature monitoring; rather, it characterizes heat severity using heat index categories (apparent temperature), classifying 41–54 °C as “Danger” and >54 °C as “Extreme danger” [15].”

Text originally stated: “Temperatures observed in Madhesh kitchens, therefore, exceed established occupational-safety thresholds by a substantial margin, rendering dehydration, renal stress, high heart rate, and blood pressure plausible daily outcomes rather than isolated events [20–22]”.

A correction has been made to the section **2 Nepal's evolving thermal landscape**, Paragraph 5:

“These regional heat index levels, therefore, exceed established occupational-safety thresholds by a substantial margin, rendering dehydration, renal stress, high heart rate, and blood pressure plausible daily outcomes rather than isolated events [20–22].”

Text originally stated: “Physiological—sustained kitchen temperatures of 50–54 °C in Dalit dwellings suppress appetite and fluid intake”.

A correction has been made to the section **5 Thermal labor, nutrition, and time poverty**, Paragraph 1:

“1. Physiological —cooking zone heat exposure suppresses appetite and can reduce fluid intake”

Text originally stated: “Direct thermal strain (50–54 °C during midsummer cooking) can suppress appetite and fluid intake.”.

A correction has been made to the section **5 Thermal labor, nutrition, and time poverty**, **Figure 2**:

“Direct thermal strain can suppress appetite and fluid intake.”

Text originally stated: “Field measurements by the Nepal Health Research Council show that kitchen air temperatures in Dalit kitchens reach 50–54 °C during midsummer meal preparation [15]”.

A correction has been made to the section *5.1 Physiological pathway*, Paragraph 1:

“Field reports by the Nepal Health Research Council indicate that regional midsummer heat indices reach “Danger” (41–54 °C) and “Extreme danger” (>54 °C) levels [15].”

Text originally stated: “Temperatures above 50 °C, therefore, constitute a clinically meaningful threat to appetite, hydration, and overall metabolic balance.”

A correction has been made to the section *5.1 Physiological pathway*, Paragraph 1:

“The compounding thermal burden of cooking in these extreme ambient conditions, therefore, constitutes a clinically meaningful threat to appetite, hydration, and overall metabolic balance.”

Text originally stated: “These findings suggest that sustained kitchen temperatures above 50 °C may directly reduce dietary intake, with disproportionate impacts on those already carrying the heaviest domestic workloads.”

A correction has been made to the section *5.1 Physiological pathway*, Paragraph 2:

“These findings suggest that sustained kitchen temperatures may directly reduce dietary intake, with disproportionate impacts on those already carrying the heaviest domestic workloads.”

Text originally stated: “Because midsummer kitchen temperatures in Dalit homes exceed 50 °C, a comparable risk of heat-driven dietary simplification likely exists in Madhesh.”

A correction has been made to the section *5.2 Behavioral pathway*, Paragraph 2:

“Given the severe compounding thermal burden of cooking during midsummer heatwaves, a comparable risk of heat-driven dietary simplification likely exists in Madhesh.”

Text originally stated: “In rural Madhesh, where electrification remains uneven, well-insulated improved biomass stoves [48] or solar–electric hybrid models [49] can reduce kitchen heat by an estimated 30–40 percent while accommodating local food practices [50].”

A correction has been made to the section **6 Discussion**, *6.1 Policy implications: measuring and mitigating thermal risk*, Paragraph 2:

“In rural Madhesh, where electrification remains uneven, well-insulated improved biomass stoves [48] or solar–electric hybrid models [49] offer improved end-use efficiency, thereby mitigating the overall thermal burden of cooking while accommodating local food practices [50]”

The original version of this article has been updated.

